# Properties of PBZTS Ferroelectric Ceramics Obtained Using Spark Plasma Sintering

**DOI:** 10.3390/ma16175756

**Published:** 2023-08-23

**Authors:** Dagmara Brzezińska, Dariusz Bochenek, Przemysław Niemiec, Grzegorz Dercz

**Affiliations:** Institute of Materials Engineering, Faculty of Science and Technology, University of Silesia in Katowice, 75 Pułku Piechoty 1 A, 41-500 Chorzów, Poland; dariusz.bochenek@us.edu.pl (D.B.); przemyslaw.niemiec@us.edu.pl (P.N.); grzegorz.dercz@us.edu.pl (G.D.)

**Keywords:** PZT material, ferroelectrics, dielectric properties, spark plasma sintering

## Abstract

In this paper, spark plasma sintering was used to obtain and investigate (Pb_0.97_Ba_0.03_)(Zr_0.98_Ti_0.02_)_1−*x*_Sn*_x_*O_3_ (PBZTS) ceramic materials for *x* = 0, 0.02, 0.04, 0.06, and 0.08. Crystal structure, microstructure, dielectric and ferroelectric properties, and electrical conductivity tests of a series of samples were carried out. The SPS sintering method ensures favorable dielectric and ferroelectric properties of PBZTS ceramic materials. X-ray studies have shown that the material has a perovskite structure. The samples have a densely packed material structure with properly crystallized grains. The fine-grained microstructure of the PZBZTS material with high grain homogeneity allows the application of higher electric fields. Ceramic samples obtained by the SPS method have higher density values than samples obtained by the classical method (FS). The permittivity at room temperature is in the range of 245–282, while at the phase transition temperature is in the range of 10,259–12,221. At room temperature, dielectric loss factor values range from 0.006 to 0.036. The hysteresis loops of PBZTS ceramics have a shape typical for ferroelectric hard materials, and the remnant polarization values range from 0.32 to 0.39 µC/cm^2^. The activation energy *E*_a_ values of the PBZTS samples result mainly from the presence of oxygen vacancies. The PZT material doped with Ba and Sn and sintered via the SPS method has favorable physical parameters for applications in modern devices such as actuators or pulse capacitors.

## 1. Introduction

PZT-type materials are obtained by various technological methods to improve their physical properties. Usually, the classical technology of obtaining ferroelectric materials begins by preparing a stoichiometric mixture of starting oxides or carbonates. In the technology process, the ceramics samples are obtained mainly by the free sintering method (FS, pressureless) [1]. Classical ceramic technology is simple and relatively cheap, but the obtained ceramic materials have an average set of parameters with high dielectric loss values. Other known and used sintering methods that change the properties of ceramics include the HP method (hot pressing method) [2], microwave sintering [1,3], spark plasma sintering (SPS) [2,4,5,6], the cold sintering process (CSP) [7,8], and flash sintering [9]. The SPS method is also known as the field-assisted sintering technique and uses uniaxial pressure and pulses of electric current (high intensity and low voltage) [6,10,11]. In the SPS method, densification of powder materials consists of heating the substance by passing pulses of electric current across its surface. Compared to traditional sintering and hot pressing methods, it enables a significant reduction in temperature and a shortening of compaction time [12]. High heating rates enable the flow of direct current through the sintered material, which prevents grain growth and positively affects the density of the material [13]. At high local temperatures, spark plasma is generated momentarily for optimal thermal and electrolytic diffusion, and a low-voltage current pulse increases the kinetics of diffusion processes [14]. Such conditions favor grain-boundary and surface diffusion; as a result, densification dominates over grain growth [15]. Analyses of the mechanisms involved during spark plasma sintering of non-conducting ceramics can be found in many works [16,17,18]. The SPS method has become helpful for the fabrication of advanced ceramics materials such as nanostructural ceramics, functionally graded materials (FGMs), metals, ceramics, ceramic matrix composite materials, amorphous alloys, and metallic glass [6,10,15,19,20,21]. For example, the possibility of obtaining dense samples of the ferroelectric BaTiO_3_ (BT) material (submicrometer size) by the SPS method was presented in [22]. Based on (AC) impedance spectroscopy measurements, authors showed that the SPS method effectively reduces the influence of intergranular (grain boundary) effects on the permittivity and DC resistance values of BT ceramics [22].

PZT-type ceramic materials have been used for a long time as sensors and actuators that transform energy and pulse capacitors. The rests of the application are ultrasonic transducers, mechanical energy harvesters, high-precision actuators, tactile sensors, and surface acoustic wave SAW devices [23,24]. One of the essential stages of ferroelectric ceramic technology is the poling process. The efficient and stable polarization of ferroelectric samples allows them to be used as piezoelectric transducers. Piezoelectric transducers are used as actuators, sensors, bimorphs, and igniters. A critical application of piezoelectrics is energy harvesters [25]. In the case of such elements, the energy is derived from external sources, e.g., the energy of vibrations, electromagnetic waves, solar power, thermal energy, and wind energy. In the energy harvesters, obtained energy is usually captured and stored for small, wireless autonomous devices, such as those used in wearable electronics and wireless sensor networks [25,26,27]. Similar applications are made of electrostrictive transducers made of relaxor materials; however, they do not need to be polarized. Ferroelectric materials exhibit several features saturated *P*-*E* hysteresis loop, high and sharp maximum permittivity at Curie temperature, and ferroelectric domains [28,29]. In turn, the relaxor materials exhibit a narrow hysteresis loop and a broad maximum of permittivity at the temperatures phase transition. In relaxors, the maximum permittivity exhibits a relatively strong dependency on frequency. Earlier, such materials were known as ferroelectrics with diffused phase transitions. Relaxor materials can be used, for example, in electrostrictive transducers (without polarization) [30,31,32].

In this paper, the (Pb_0.97_Ba_0.03_)(Zr_0.98_Ti_0.02_)_1−*x*_Sn*_x_*O_3_ (PBZTS) materials were obtained using the SPS technology, where *x* = 0, 0.02, 0.04, 0.06 and 0.08. This paper aims to investigate how the SPS technology influences the ferroelectric and dielectric properties of PBZTS materials compared with the classical method. In our previous papers, PBZTS materials have been obtained using classical ceramic technology, e.g., [33,34,35]. The results show that ceramic PBZTS samples have an ideal microstructure with low porosity and clearly defined grain boundaries and exhibit high dielectric permittivity values [35]. The SPS technology can improve the ferroelectric and dielectric properties of the materials. Improving the properties of materials may allow obtaining efficient transducers of smaller sizes in the future, which is very important in modern applications.

## 2. Experimental

The PBZTS powders prepared for classical sintering technology were used for this experiment. The following reagents were used in the technological process, i.e., BaCO_3_ (99.99%, POCH, Gliwice, Poland), PbO (99.9%, POCH, Gliwice, Poland), TiO_2_ (99.9%, Merck, Darmstadt, Germany), ZrO_2_ (99.5%, Aldrich, St. Louis, MO, USA), and SnO_2_ (99.9%, Aldrich, St. Louis, MO, USA). The powders were weighed according to the stoichiometric ratio and mixed in the planetary ball mill (FRITSCH Pulverisette 6, Idar-Oberstein, Germany) for 15 h in ethanol. Excess PbO (5%) was used during the technology. In the next stage, the powders were calcined at *T*_calc_ = 850 °C/*t*_calc_ = 3 h and milled again. In the case of synthesizing perovskite materials, too low temperatures and too short times of the synthesis process are the most common reason for the formation of a non-single phase. The PbZr_1−*x*_Ti*_x_*O_3_ solid solution is formed as a result of complex chemical reactions. It is formed gradually with the temperature increase, and the synthesis process practically ends at approximately a temperature 800 °C [36].

**Figure 1:**
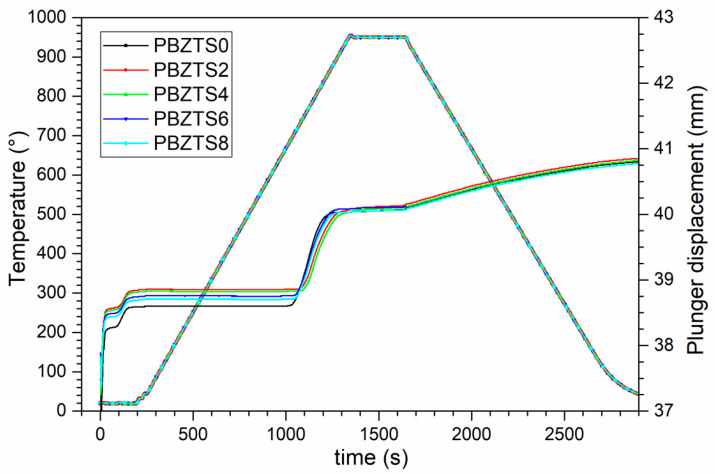
Plots of the SPS process’s temperature rise and plunger displacement for PBZTS ceramic materials.

PBZTS powders synthesized under the abovementioned conditions were used for sintering by the SPS spark plasma sintering method. The process of sintering the PBZTS material was carried out in an argon atmosphere under the conditions of temperature (950 °C), dwell time (5 min), pressure (50 MPa), heating rate 50 °C/min, pressure (50 MPa). The sintering conditions in the SPS method were selected based on literature data [6,10] and our previously conducted experimental studies related to the preparation of multi-component ceramic materials and the parameters of the densification degree recorded by the SPS device. In the SPS process of ceramic materials, many technological parameters are recorded, e.g., temperature, time, rate of heating and cooling, and piston pressure. Changing the piston’s position allows for determining the compaction curve based on the current baling pressure and temperature (the change in the specific volume of the powder corresponds to the change in the position of the piston). Figure 1 shows charts of the SPS process’s temperature rise and plunger displacement for PBZTS ceramic materials. After sintering, the ceramic samples were grinding, polishing, and annealing to remove mechanical stresses. For electric tests, the silver paste was applied on the surfaces of the samples (firing method at 850 °C/15 min). The chemical compositions of the analyzed PBZTS samples and their abbreviated designations are summarized in Table 1.

The relative density of the PBZTS ceramic materials was estimated using the Archimedes method. XRD measurements were conducted using a Philips diffractometer in the angular range from to (filtered radiation CuK_α_, deg. step 0.04). The analysis of surfaces (EDS) and SEM microstructure of the ceramic samples was performed using Jeol scanning electron microscope (JSM-7100 TTL LV). The average grain size was measured using the ImageJ program. The dielectric measurements were performed using the QuadTech LCR in the 20 to 420 °C temperature range and at frequencies from 500 Hz to 1.0 MHz (heating cycle). Hysteresis loops *P*-*E* were measured at room temperature using a high-voltage amplifier (Matsusada Precision Inc., Kusatsu, Japan, HEOPS-5B6) and a Sawyer-Tower circuit. An International Instruments NI USB-6002 digital card captured the data. DC electrical conductivity was measured using the Keithley electrometer (6517B) in the temperature range of 20–420 °C, while AC electrical conductivity was calculated from the dielectric measurements. The relationship between AC conductivity and frequency was determined according to Jonscher’s universal power law (UDR).

## 3. Results and Discussion

**Figure 2:**
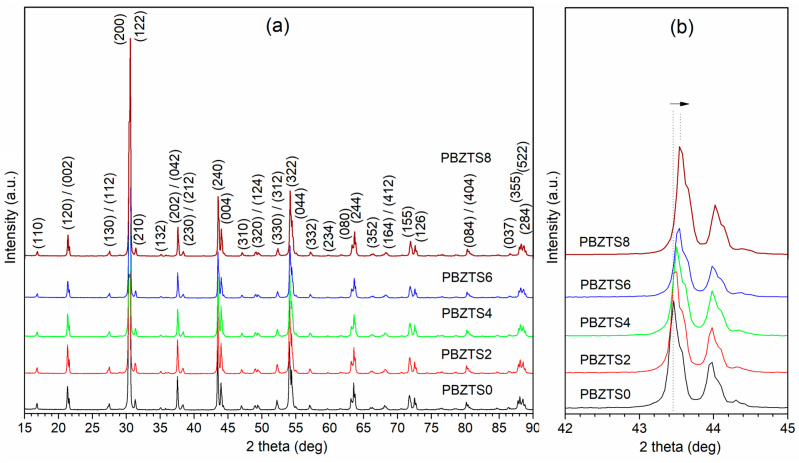
The X-ray diffraction patterns of PBZTS ceramics at room temperature (**a**) and enlarge range fragment for (240)/(004) lines (**b**).

The X-ray diffraction patterns of the PBZTS ceramic materials are depicted in Figure 2. X-ray measurements have shown that all the diffraction lines of the PBZTS samples correspond to the perovskites phase (matching was performed according to the PbZrO_3_ pattern, i.e., ICDD PDF01-089-8012, crystal system orthorhombic, space group P*nnm*) [38]. X-ray analysis confirmed the absence of foreign phases in the PBZTS compositions. Work [39,40] presents the phase diagram of the PZT material whose crystalline structure changes with the change in the Zr/Ti content. The orthorhombic structure of the PZT-type material (for small Ti concentration) is more complex, i.e., there are numerous reflections in the diffraction pattern. Similar research results were presented in [38,41,42], where a detailed analysis of the crystal structure for a wide range of PBZT-type compositions was made. However, in [33], it was shown that the increase in tin’s content reduces the unit cell’s size. It may be connected to the fact that the ionic radius of Sn^4+^ (0.074 nm) is smaller than the Zr^4+^ (0.087 nm). In the case of titanium, the ion is smaller (0.061 nm), but in the analyzed materials, the amount of Ti in the chemical composition is meager. Figure 2b shows an enlarged fragment of the X-ray diagram of PBZTS materials for reflections (240) and (004) belonging to the orthorhombic phase. The graph shows a shift of reflections towards higher angles for the composition with increasing amounts of tin. It may indicate a decreased distance between atoms in the unit cell.

**Figure 3:**
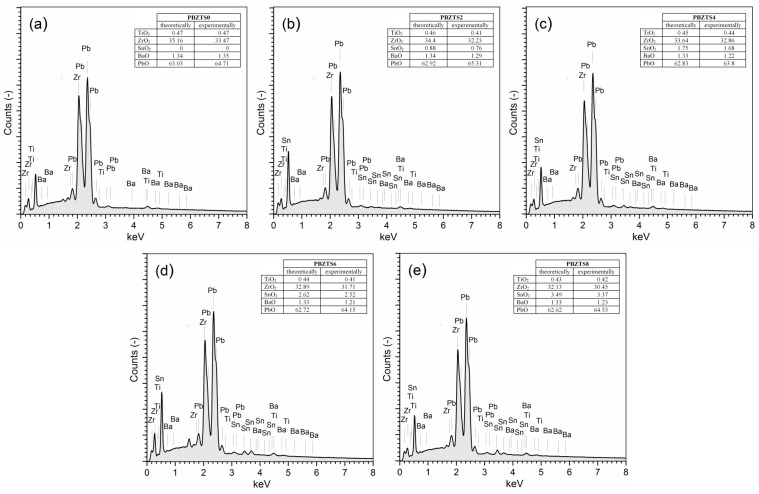
The EDS analysis image of the element distribution for the PBZTS ceramic samples: PBZTS0 (**a**), PBZTS2 (**b**), PBZTS4 (**c**), PBZTS6 (**d**), and PBZTS8 (**e**).

The PBZTS ceramic samples obtained by the SPS method have a high density and significantly exceed the density values of the samples obtained by the classical method (Table 2) [37,43]. The surface EDS test was performed on a large sample area. The results of the surface EDS analysis are the average of five measurements made on the surface microstructure of the sample. The results of the EDS analysis are presented in the tables in Figure 3. The analysis showed the absence of foreign elements and impurities, a slight excess of lead oxide, and a deficit of ZrO_2_, TiO_2_, SnO_2_, and BaO. It may be due to the PbO addition used during the synthesis of the PBZTS material.

**Figure 4:**
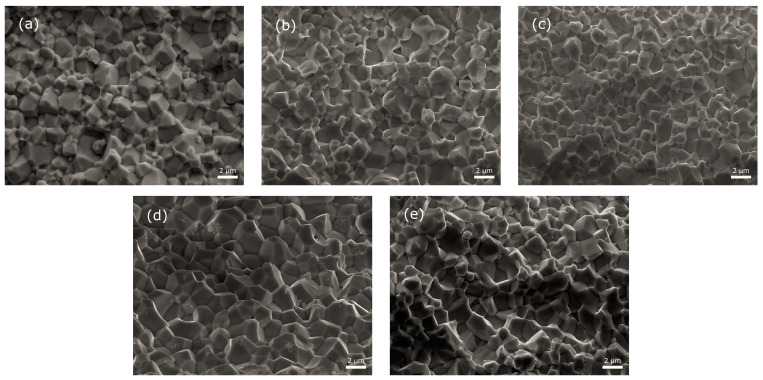
Microstructure of PBZTS ceramic samples obtained using SPS technology: PBZTS0 (**a**), PBZTS2 (**b**), PBZTS4 (**c**), PBZTS6 (**d**), and PBZTS8 (**e**).

**Figure 5:**
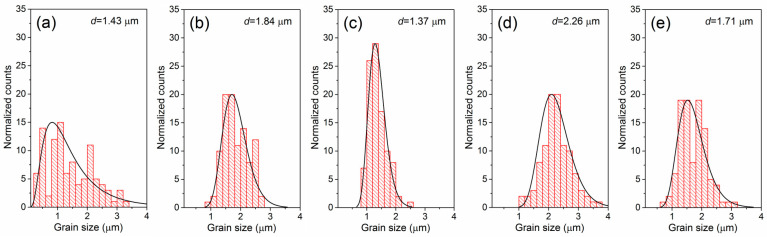
Statistical results of the grain size distribution of PBZTS ceramic samples: PBZTS0 (**a**), PBZTS2 (**b**), PBZTS4 (**c**), PBZTS6 (**d**), and PBZTS8 (**e**).

The microstructural images of a cross-section of the surface PBZTS samples are shown in Figure 4. All ceramic samples show well crystallized and fine-grained microstructure, with an average grain size of 1.37 to 2.26 μm. The results of the grain size distributions of all samples are presented in Figure 5. In the microstructure of PBZTS0, there are both fine and larger grains (high inhomogeneity of grains in the microstructure). This inhomogeneity is because, with the temperature increase in the ceramic microstructure of the sample, large grains grow faster at the expense of small grains. The SEM images (Figure 4) and distribution diagrams of average grain sizes (Figure 5) show that the Sn-doped PBZT compositions (PBZTS) show an increase in grain homogeneity in the microstructure but no clear trend of changes depending on the amount of Sn in the composition is observed. PBZTS2 and PBZTS4 show a smaller average grain size, while PBZTS4 and PBZTS6 show the most excellent homogeneity. Increasing the amount of Sn (above *x* = 0.06) shows no further improvement in said microstructure parameters. In paper [35], it was shown that the amount of Sn admixture is of great importance for the properties of PBZT materials, i.e., the excessive tin doping of the PBZT materials can lead to an increase the structural defects and lattice stress, causing deterioration of elecro-physisal (dielectric and ferroelectric) parameters. The classical method does not always allow obtaining a ceramic material with a properly crystallized grain. In these cases, the grains have blurred boundaries (rounded shapes) and high porosity, presented by the paper’s authors [38] for the PBZT material with a similar chemical composition.

**Figure 6:**
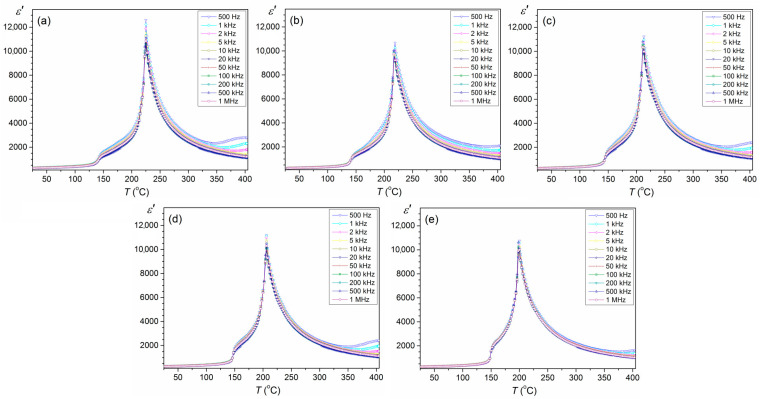
Permittivity vs. temperature of PBZTS ceramic samples: PBZTS0 (**a**), PBZTS2 (**b**), PBZTS4 (**c**), PBZTS6 (**d**), and PBZTS8 (**e**).

In Figure 6, the results of measurements of permittivity versus temperature are depicted (heating cycles). For the entire sample series, the temperature dependencies of permittivity exhibit two characteristic anomalies. The first occurs approximately 150 °C and is associated with the ferroelectric-paraelectric phase transition. With the increase in the tin admixture, this anomaly is shifted towards higher temperatures. The second maximum permittivity is approximately 200–225 °C, related to the ferroelectric-paraelectric phase transition (*T*_m_). Similar results were obtained in work [43] for PBZT ceramics sintered by the solid-state reaction method but with much lower maximum permittivity values and at room temperature. Additionally, comparing the dielectric properties of samples obtained by two methods, i.e., SPS and FS [37], it can be concluded that the SPS method significantly improves permittivity values (Table 2). In the case of the maximum permittivity (at *T*_m_) a slight decrease in the permittivity value with increasing frequency occurs. The frequency dispersion temperature phenomena shift the maximum permittivity values *ε*(*T*) towards higher temperatures with increasing frequency. For 1 kHz, there is the highest permittivity value at *T*_m_ for undoped PBZTS0 ceramics, i.e., 12,221. It can be seen that *T*_m_ decreases with increasing the content of Sn, and permittivity values are lower than for PBZTS0 (see Table 2).

**Figure 7:**
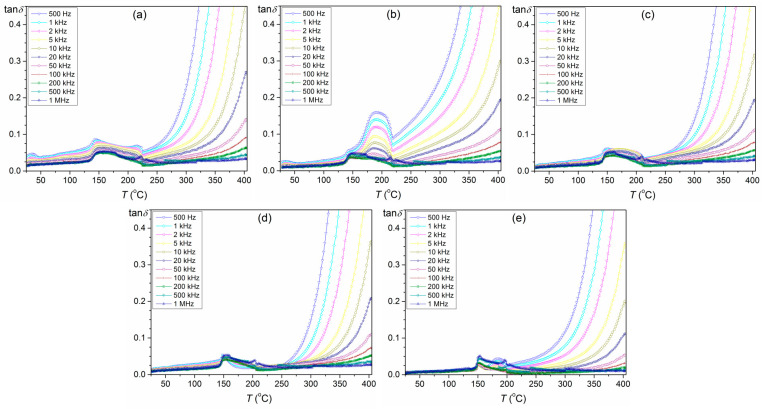
Dielectric loss factor vs. temperature of PBZTS ceramic samples: PBZTS0 (**a**), PBZTS2 (**b**), PBZTS4 (**c**), PBZTS6 (**d**), and PBZTS8 (**e**).

In Figure 7, dielectric loss is shown in terms of the dielectric loss factor (tan *δ*). As in the *ε*(*T*) plots, there are also two maxima on the temperature dependencies of tan *δ*. In the case of PZT ceramics, there is a noticeable (local) decrease in dielectric loss values just before the phase transition. The dielectric loss factor values decrease with increasing frequency, while above 225 °C, their values increase rapidly with increasing temperature. No clear relationship was observed between the increase in the Sn value and the shape and location of the maximum dielectric loss factor. Additionally, significantly lower dielectric loss factor values are obtained for the PBZTS materials sintered by the SPS method compared to the classical method (Table 2).

**Figure 8:**
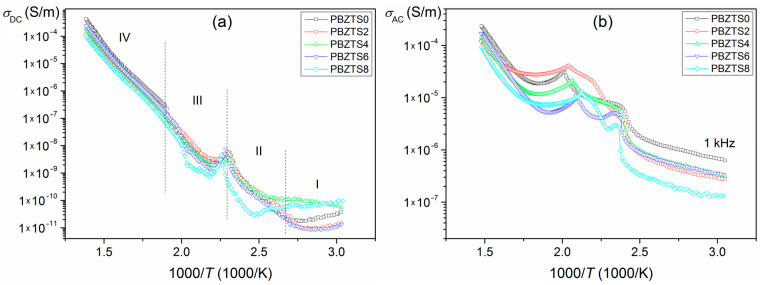
DC electrical conductivity measured at 100 V (**a**) and AC electrical conductivity of the PBZTS ceramic samples calculated from Formula (1) (**b**).

The results of the DC electrical conductivity (*σ*_DC_) and the calculations of AC electrical conductivity (*σ*_AC_) for the analyzed samples are presented in Figure 8. The activation energy was calculated from rectilinear sections of the graph *σ*(1000/*T*) in four temperature ranges (Table 3). In the case of the DC conductivity, the activation energy in area I is in the range 0.09–0.06 eV, in area II is in the range 0.24–0.74 eV, in area III is in the range 0.21–0.53 eV, and in area IV, is in the range 0.51–0.52 eV. The activation energy *E*_a_ values calculated in the I, II, and III areas are mainly governed by the ionization processes, and electrons or holes are the prevailing charge carriers [44]. Slightly higher activation energy values occur in the IV range, associated with increased electrical conductivity of the ceramic samples at a higher temperature. In this case, the conductivity mechanism is associated with extrinsic defects and, more precisely, with these defects’ increased mobility [44]. The obtained activation energy results correspond to the values for most ferroelectric materials, for which electrical conductivity is related to oxygen vacancies and dipolar defect structure [45,46].

Based on the dielectric measurements and the formula below, the AC electrical conductivity (Figure 8b) was calculated:

(1) $$\sigma_{AC}=\omega \varepsilon_{0}\varepsilon^{\prime }tan\delta$$

where *ω* is the angular frequency (*ω* = 2π*f*), *ε*_0_ is the value of the absolute dielectric permittivity of classical vacuum, *ε*′ is the real-valued permittivity, and tan *δ* is the dielectric loss factor.

The test results show that the AC electrical conductivity values are higher than that of DC electrical conductivity ones. In the areas of the phase transition temperature (Figure 8b, 1 kHz), considerable local maxima of the AC conductivity have been observed, while in the temperature ranges far from the Curie temperature, such anomalies are not observed. It means that the influence of ferroelectric properties on the conductivity in these ranges is less pronounced. Similar research results were observed in work [46] for the PZT-type material, where it was also shown that variable-range hopping is realized at low temperatures. An electron prefers to jump to a more remote site than to the nearest-neighbor one to reduce the energy required from the hop. On the other hand, at high temperatures (above the phase transition), AC conductivity shows an activated character [46].

**Figure 9:**
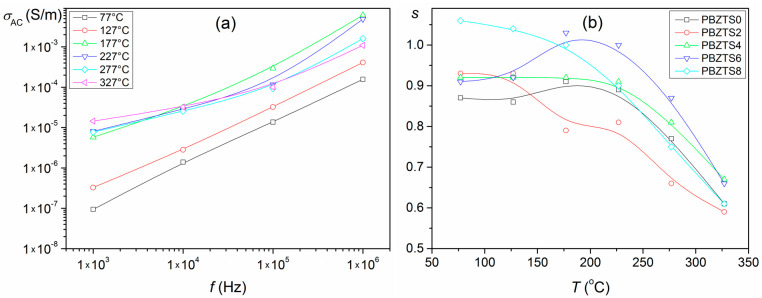
AC conductivity as a function of frequency for PBZTS8 sample (**a**) and the *s*(*T*) relationships obtained by fitting experimental results to Jonscher’s law for PBZTS materials (**b**).

Jonscher’s universal power law (UDR) describes the relationship between AC conductivity and frequency [47,48]:

(2) $$\sigma_{AC}\left(\omega \right)=A\omega^{s}$$

where *σ*_AC_(*ω*) is the AC conductivity, *A* is a constant, *ω* is the angular frequency, and *s* is the frequency exponent parameter—dependent on temperature and independent of frequency. For the PBZTS ceramics, AC conductivity was determined according to Jonscher’s law (UDR), while the PBZTS8 ceramic sample is presented in the frequency graph in Figure 9a, as representative. Based on the abovementioned graph, two different conductivity mechanisms can be distinguished. The first conductivity mechanism represents the relativity weak temperature dependence and is connected to the hopping of charge carriers, while the second conductivity mechanism shows the firm temperature dependence component [49]. Based on the frequency *σ*_AC_ curves, the *s* parameter was calculated. Figure 9b presents the experimental *s* parameter values calculated for all PBZTS compositions. The temperature dependence of the *s* parameter can determine the suitable mechanism involved for AC conductivity [42]. The frequency-dependent AC conductivity fullfils the UDR power law—Formula (2). This parameter is generally considered to be between zero and unity, although there are examples where *s* > 1 (~1.03–1.05, especially at relatively low temperatures). The *s* parameter is also seen as temperature dependent. At lower temperatures, the exponent *s* is higher and decreases with increasing temperature (PBZTS0 and PBZTS6 samples show some deviation). For example, the *s* parameter decreases when the temperature rises from 0.87 to 0.61 for the PBZTS0 composition and from 1.06 to 0.61 for the PBZTS8 composition. The value of *s* close to one at low temperatures indicates that AC conductivity is predominant due to the relaxation dipole movement [50]. Similar trends are observed in crystalline materials, amorphous semiconductors, and glasses [50,51,52].

The most probable defect in perovskite materials is oxygen vacancies [46]. When these defects appear in the structure, two Ti^5+^ ions will be found to maintain the electrical neutrality, and around an oxygen vacancy, the long-range potential well is formed. The oxygen vacancy may affect the localization energy at Ti and Zr sites. In this case, two types of carrier movement can be distinguished, i.e., hopping between Ti or Zr sites contained within one defect potential well and hopping of electrons between Ti^4+^ (Zr^4+^) to Ti^5+^ (Zr^5+^) sites. Because the average distance a carrier passes is the mean distance between oxygen vacancies, the DC conductivity may be associated with the hopping between the long-range potential well created by oxygen vacancies. In contrast, AC conduction can occur through carrier motion over a shorter range distance between sites in the potential well [46].

**Figure 10:**
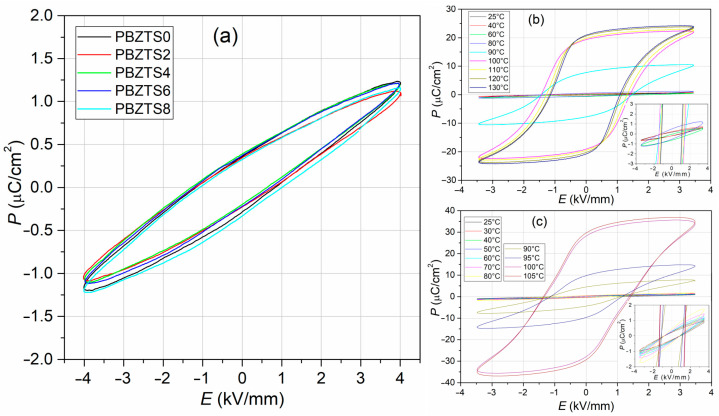
*P*-*E* hysteresis loops of PBZTS ceramic samples, at room temperature (**a**), temperature *P-E* loop for PBZTS6 sample sintered via the FS method (**b**) and for PBZTS2 sample sintered via the SPS method (**c**).

*P*-*E* hysteresis loops at room temperature for all PBZTS samples are shown in Figure 10. The analyzed compositions show hysteresis loops characteristic for materials of medium ferroelectric hardness, with weak saturation at the field of 4 kV/mm. The Sn admixture introduced to the base material does not significantly change the shape of the *P*-*E* loop. The remnant polarization (*P*_r_) values are low, i.e., 0.36 µC/cm^2^ (PBZTS0), 0.35 µC/cm^2^ (PBZTS2), 0.39 µC/cm^2^ (PBZTS4), 0.37 µC/cm^2^ (PBZTS6), 0.32 µC/cm^2^ (PBZTS8), whereas the coercive field (*E*_c_) values are 1.01, 0.99, 0.96, 0.97, 1.04 kV/mm, for PBZTS0, PBZTS2, PBZTS4, PBZTS6, and PBZTS8, respectively. However, no clear trend of changes in ferroelectric parameters with increased Sn amount in the PBZTS composition was observed. The highest value of *P*_r_ and the lowest value of *E*_c_ shows the composition PBZTS4. The values of the remnant polarization, maximum polarization, the coercive field, and other electro-physical parameters for PBZTS ceramics obtained by both the SPS and FS methods are presented in Table 2. The SPS method increases the tested samples’ remnant and maximum polarization values while the coercive field values are lower.

Figure 10b,c shows the change in polarization of PBZTS samples with increasing temperature for exemplary samples, i.e., PBZTS6 sintered via the FS method and PBZTS2 sintered via the SPS method (inside *P-E* plots up to 80 °C). At room temperature, the PBZTS samples show low *P*_r_ polarization values; however, with increasing temperature, there is a significant increase in the remnant and maximum polarization values. Above 90 °C, the growth of *P*_r_ is rapid. According to the PZT phase diagram presented by Jaffe [40] for low Ti contents, a phase transition (orthorhombic/rhombohedral) is observed in PZT with increasing temperature. Generally, at room temperature, the domain structure of the orthorhombic phase is quite complex [53], and the spontaneous polarization can be oriented along multiple crystallographic axes. There are also domains arranged perpendicularly to the applied field, whose reorientation is inefficient and requires higher fields. It contributes to low polarization values. At higher temperatures, the domain structure of PBZTS samples transforms (to rhombohedral arrangement), and the domains quickly reorient to the direction of the electric field, contributing to the increase in polarization [54]. On the other hand, the coercive field values decrease, facilitating the domain reorientation process. Similar changes in *P*_r_, *P*_m_, and *E*_c_ with increasing temperature were observed at work [33,54].

## 4. Conclusions

This work aimed to obtain via the spark plasma sintering method and measurements of the (Pb_0.97_Ba_0.03_)(Zr_0.98_Ti_0.02_)_1−*x*_Sn*_x_*O_3_ (PBZTS) materials for different *x* = 0, 0.02, 0.04, 0.06, and 0.08. Thanks to the use of lower energy in the technological process, the SPS is an effective sintering method that allows obtaining PBZST materials with a fine-grained microstructure and favorable final properties, in a much shorter time and at a lower temperature. X-ray studies have shown that the material has a perovskite structure. The PBZTS samples have a densely packed material microstructure with properly crystallized grains, which is more excellent than for samples obtained in the classical sintering method. The admixture of tin preferably influences the microstructure of the PBZTS samples, i.e., improves grain homogeneity. The PBZTS4 and PBZTS6 samples have the most excellent homogeneity, whereas PBZTS2 and PBZTS4 show a smaller average grain size. Increasing the amount of Sn above *x* = 0.06 is unfavorable and results in the deterioration of these parameters. At room temperature, the permittivity is in the range of 245–282, while dielectric loss factor values are in the range of 0.006–0.036. Comparing the properties of the PBZTS material obtained by the classical and the SPS methods, the samples have the correct microstructure with higher grain uniformity and density in the SPS method. It is conducive to improving the PBZTS materials’ physical properties, including dielectric properties, i.e., obtaining higher permittivity values at room temperature and the phase transition temperature and lower dielectric loss values. Additionally, higher ferroelectric properties of the PBZTS material can be obtained using the SPS method. The shape of the hysteresis loops of the PBZTS materials is typical for ferroelectric hard materials. The remnant polarization values range from 0.32 to 0.39 µC/cm^2^, the spontaneous polarization values range from 0.42 to 0.56 µC/cm^2^, and the coercive fields range from 0.96 to 1.04 kV/mm. The activation energy *E*_a_ values are from 0.02 to 0.74 eV, suggesting oxygen vacancies in the PBZTS ceramic samples. In the PBZTS materials, the DC conductivity may be associated with the hopping between the long-range potential well created by oxygen vacancies. In contrast, AC conduction can occur through the carrier motion between sites in the potential well.

The obtained test results of the PBZTS material indicate its potential applications for electromechanical transducers, such as energy harvesters and sensors.

## Figures and Tables

**Table 1:** Chemical compositions of the analyzed series of PBZTS samples (according to [37]).

Abbreviation	Basic Composition Mark	*x* (Sn Content)
PBZTS0	Pb_0.97_Ba_0.03_(Zr_0.98_Ti_0.02_)O_3_	0
PBZTS2	Pb_0.97_Ba_0.03_(Zr_0.98_Ti_0.02_)_0.98_Sn_0.02_O_3_	0.02
PBZTS4	Pb_0.97_Ba_0.03_(Zr_0.98_Ti_0.02_)_0.96_Sn_0.04_O_3_	0.04
PBZTS6	Pb_0.97_Ba_0.03_(Zr_0.98_Ti_0.02_)_0.94_Sn_0.06_O_3_	0.06
PBZTS8	Pb_0.97_Ba_0.03_(Zr_0.98_Ti_0.02_)_0.92_Sn_0.08_O_3_	0.08

**Table 2:** The electro-physical parameters of the PBZTS ceramic materials sintered via the FS method (according to [37]) and SPS method.

Parameter	PBZTS0	PBZTS2	PBZTS4	PBZTS6	PBZTS8
classical technology (fee sintering)					
*ρ* (g/cm^3^)	6.76	6.52	5.76	6.24	7.01
$\bar{r}$*r* (μm)	1.79	2.84	2.05	2.02	1.91
*ε*_r_ ^a,b^	134	146	156	161	166
tan *δ* ^a,b^	0.036	0.014	0.027	0.047	0.011
*T*_m_ (°C) ^b^	225	218	211	203	199
*ε*_m_ at *T*_m_ ^b^	663	1481	1168	1536	3264
tan *δ* at *T*_m_ ^b^	0.139	0.284	0.110	0.063	0.273
*P*_r_ (μC/cm^2^) ^a^	0.19	0.24	0.18	0.18	0.29
*E*_c_ (kV/mm) ^a^	1.27	1.30	0.76	1.05	1.35
*P*_max_ (μC/cm^2^) ^a^	0.49	0.65	0.79	0.63	0.71
spark plasma sintering					
*ρ* (g/cm^3^)	7.81	7.54	7.68	7.48	7.69
$\bar{r}$*r* (μm)	1.43	1.84	1.37	2.26	1.71
*ε*_r_ ^a,b^	282	245	262	263	258
tan *δ* ^a,b^	0.036	0.020	0.015	0.015	0.006
*T*_m_ (°C) ^b^	225	219	213	206	200
*ε*_m_ at *T*_m_ ^b^	12,221	10,259	10,946	11,122	10,563
tan *δ* at *T*_m_ ^b^	0.049	0.070	0.033	0.018	0.020
*P*_r_ (μC/cm^2^) ^a^	0.36	0.35	0.39	0.37	0.32
*E*_c_ (kV/mm) ^a^	1.01	0.99	0.96	0.97	1.04
*P*_max_ (μC/cm^2^) ^a^	1.23	1.12	1.21	1.22	1.16

^a^—at room temperature, ^b^—at 1 kHz.

**Table 3:** The values of the activation energy for PBZTS ceramic samples.

Parameter	PBZTS0	PBZTS2	PBZTS4	PBZTS6	PBZTS8
*E*_a_ in I (eV)	0.09	0.05	0.06	0.02	0.06
*E*_a_ in II (eV)	0.48	0.36	0.24	0.36	0.74
*E*_a_ in III (eV)	0.53	0.48	0.46	0.43	0.21
*E*_a_ in IV (eV)	0.52	0.51	0.52	0.52	0.51

## Data Availability

Data are contained within the article.
